# Effect of Technological Factors on the Extraction of Polymeric Condensed Tannins from Acacia Species

**DOI:** 10.3390/polym16111550

**Published:** 2024-05-30

**Authors:** Zeinab Osman, Antonio Pizzi, Mohammed Elamin Elbadawi, Jérémy Mehats, Wadah Mohammed, Bertrand Charrier

**Affiliations:** 1 Institute of Engineering Research and Materials Technology, National Center for Research, Khartoum P.O. Box 2404, Sudan; wdalbadawi@yahoo.co.uk (M.E.E.); wadah.eng@yahoo.com (W.M.); 2 ENSTIB-LERMAB, University of Lorraine, 27, Rue Philippe Seguin, 88000 Epinal, France; 3 University of Pau and the Adour Region, E2S UPPA, CNRS, Institute of Analytical Sciences and Physico-Chemistry for the Environment and Materials-Xylomat (IPREM-UMR5254), 40004 Mont de Marsan, France; jeremy.mehats@univ-pau.fr (J.M.); bertrand.charrier@univ-pau.fr (B.C.)

**Keywords:** tannin extraction, green methods, fraction size, bioadhesives, *Acacia nilotica*, *Acacia seyall* var. *seyall*, commercial application

## Abstract

The aim of this research work was to investigate the influence of parameters such as particle size, mass/solvent ratio, temperature and spray drying on the tannin extraction process in order to develop cost-effective methods with better environmental and structural performance. The pods of *Acacia nilotica* ssp. *tomentosa* (ANT) were fractionated into three fractions, coarse fraction (C) (>2 mm), medium fraction (M) (1–2 mm), and fine fraction (F) < 1 mµ), and extracted with different water-to-pod ratios (2:1, 4:1 and 6:1) at different temperatures (30, 50 and 70 °C). The best results were scaled up using the three fractions of ANT, its bark and the bark of *Acacia seyal* var. *seyal* (ASS). Part of their extract was spray dried. The tannin content and total polyphenolic materials were evaluated using standard methods. Their adhesives were tested for their tensile strength. Tannins of ASS were characterized by ^13^C NMR and MALDI-TOF. The results revealed that the fine fraction (F) gave the highest percentage of tannins in both small and scaled-up experiments. The results of the tensile strength conformed to the European standard. The ^13^C NMR spectra of ANT and ASS showed that the bark contained condensed tannins mainly consisting of procyanidins/prodelphinidin of 70%/30% and 60%/40%, respectively. MALDI–TOF spectra confirmed the results obtained by ^13^C NMR and detailed the presence of flavonoid monomers and oligomers, some of which were linked to short carbohydrate monomers or dimers.

## 1. Introduction

Tannins are broadly categorized into two main groups, i.e., condensed tannins and hydrolyzable tannins [1]. Being abundant in nature, the amounts of tannins in plants depend on the geography, biological origin, species, populations, age and position in the tree, i.e., bark, pods, or leaves [2,3]. In recent years, the production of tannins has become a significant issue because of their broad range of applications including tanning animal hides, medicinal uses, and use in the food industry, which have increased commercial interest in them. More than 90% of the total world industrial extraction of tannins (>220,000 tons per year) at present is composed of polyflavonoid tannins. However, the number of tannin-producing factories is still limited in spite of the huge potential for their worldwide extraction from a variety of sources that may amount to millions of tons [4]. Due to the heterogeneous nature of tannins, the extraction process remains the main challenge for their valorization and industrial utilization [5]. Industrially, condensed tannins are obtained by soaking the crushed part of the plant containing the tannins; small percentages of sodium sulfite, metabisulfite and/or sodium bicarbonate can be added to improve the solubility of tannin oligomers with a higher molecular weight to increase the percentage extraction yield. However, sulfitation, if performed excessively, is harmful depending on the end use of the tannins [6]. In laboratories, many methods have been tried; the simplest is solid/liquid extraction, where solvents with different relative polarities may be used, such as water, ethanol, acetone, or methanol. Such solvents (acetone and hexane) remain in the residues of the extraction process and represent an environmental threat that can directly affect both human and animal health threats [7]. Some of the methods with a low environmental impact and short period of extraction used for tannin extraction include supercritical solutions, pressurized water, and ultrasonication. Conversely, the major drawback of these methods is the dependance on using expensive equipment, the amount of solvent used, long time requirements, and low extraction yields. Therefore, it is necessary to explore efficient extraction methods for tannins [8,9,10,11]. Most of the reviewed methods on tannin extraction used harmful solvents or expensive techniques. Furthermore, it is necessary to keep researching so that a favorable benefit/harm balance can be achieved [7]. There remains a large gap in knowledge on extraction technology and parameters such as solvents, fraction size of the extracted materials and effect of spray drying on the amounts of condensed tannins. These factors constitute a crucial point for their reuse, valorization and sustainable production; they have not been researched comprehensively in one study. On the other hand, the potential amounts of tannins that can be extracted yearly amount to millions of tons. This large potential has not yet been realized. Many solvents have been used for the extraction of tannins such as water, organic solvents, or a mixture of water and organic solvents [12,13,14]. In spite of the fact that water extraction is economic and has a positive environmental image, it is believed that the extract usually contains phenolic monomers, mineral substances, and carbohydrates, which makes its structural elucidation difficult [15].

In Sudan, there are many indigenous plant species that contain tannins in different quantities. Acacia species were primarily found to consist of high tannin percentages [16]. The most important tree was *Acacia nilotica* subsp. *tomentosa* (ANT) and *Acacia seyal* var. *seyal* (ASS). Both trees spread naturally in the central belt of the low-rainfall savannah, where they exist in pure or mixed stands, dominating in the central part of Sudan. Previous studies revealed that their pods and barks contained good percentages of tannins [17,18]. Tannins are used for leather tanning and in traditional medicine as they possess antibacterial and antiviral properties [19,20]. *A. seyal* var. *seyal* trees cover an area of 36,000 square kilometers (3.6 million ha). This tree species is the second-highest producer of gum Arabic after *Acacia Senegal* in Sudan [21], and the wood is used extensively to produce charcoal, leaving a huge amount of the bark as waste [22]. In this study, tannin extraction using water at different temperatures and different fraction sizes was extensively studied using the pods of *Acacia nilotica* subsp. *tomentosa* (ANT). To manifest the efficiency of water extraction, the tannins extracted from bark of *Acacia seyal* var. *seyal* (ASS) were analyzed using ^13^C NMR and MALDI-TOF in order to elucidate its structure and polymeric composition.

The aim of this study was to propose an economically and environmentally friendly method for tannin extraction using only water, without any added chemicals. It also aims to elucidate the effect of certain technological parameters, such as the fraction size of tannin-containing materials, solvent/mass ratio, and extractive temperature, on the chemical composition and structure of tannins that have not previously been comprehensively studied.

## 2. Materials and Methods

### 2.1. Materials

The pods and bark of ANT were selected randomly from the floor of a compartment of a 10-year-old “Sunt” stand in Hariri Forest (Sinnar State, Sudan). The bark of ASS was collected from a forest in the Blue Nile state (Sudan); fours trees were selected randomly, felled and debarked, and then the bark was air dried and kept in plastic bags prior to further processing.

### 2.2. Methods

The samples were air-dried before a portion of the pods (11 kg) was slightly crushed using a wooden mortar without damaging the seeds, and they were then divided by sieving into coarse fraction (C), retained on a mesh 10 (>2 mµ) sieve and medium fraction (M) (1–2 mµ), which passed through a 10 mµ mesh. Another portion of the pods (6 kg each) and the bark of *Acacia seyal* var. *seyal* were reduced to a fine powder (F < 1 mµ) using a star mill. 

#### 2.2.1. Small-Scale Experiments

Three series of aqueous tannin extraction from pods were carried out. For the extraction of tannins, aliquot portions of 200 g each of ground-pod fractions (C, M and F) were soaked in water at three different initial temperatures (30, 50, and 70 °C), in three different water-to-pod ratios (2:1, 4:1 and 6:1), for overnight extraction period. The obtained extracts were filtered using a special cloth. As a result of the above extraction, 27 extracts were obtained, coded according to the fraction particle size, initial temperature of the extraction water, and water-to-pod ratio. 

#### 2.2.2. Large-Scale Experiments

An amount of 5 kg was taken from each of the three fractions (F, M and C) of the ground pods as well as from the ground bark of ANT and ASS, which was soaked in 30 L of water with a 70 °C initial temperature for each. The extracts were filtered using special cloth. The filtrates were fed into an evaporator using a steam temperature ranging between 68 and 80 °C, under 167 to 526 KPa of pressure for 45–120 min. 

#### 2.2.3. Spray Drying

The filtrates of the tannins were spray dried using a laboratory spray dryer (Bowe Engineering, Carlow, Ireland) at 175 °C and a flow rate of 10 mL/min.

#### 2.2.4. Chemical Analysis

The aqueous tannin solutions with a 40% concentration were prepared from all tannin extracts; liquid and solids from the pods and bark of ANT and ASS (bark only) were analyzed for their tannin content, non-tannins, total solids, total solubles, and tannin purity using official hide powder standard methods [23]. 

##### Determination of Stiasny (Catechin) Number

A measure of 100 mL of the tannin aqueous solution (L) was filtered on crucible G4; the filtrate was reacted with Stiasny reagent (5 mL HCL + 10 mL of formaldehyde at 37%). The mixture was maintained for 24 h at room temperature; then, the solution and the precipitate were filtered, and the precipitate (C) was dried at 105 °C until it reached a constant mass. The percentage of the total phenolic materials (precipitate) was calculated according to the equation described by Yazaki et al. [24].

#### 2.2.5. Preparation of the Resins

Tannin-based adhesives were prepared from the spray-dried powder and concentrates of aqueous tannin extracts of the three fractions and Mimosa of the large-scale experiments. To the aqueous solutions with 40% tannin concentrations, 5% paraformaldehyde was added.

#### 2.2.6. Resin Testing

Testing was carried out in accordance with DIN 52186 (1978) [25]. Beach wood veneer pieces 20 × 20 × 300 mm in size with moisture contents of 5% were tested. The glued specimens were further tested with a universal tester for tensile strength to calculate their adhesive strength. 

#### 2.2.7. ^13^C NMR Analysis

The concentrated tannin extracts (aqueous solution 47%) were analyzed using ^13^C NMR according to a previously reported method [26]. The liquid ^13^C-NMR spectrum of the tannin extracts was obtained on a Bruker MSL 300 FT-NMR spectrometer (Bruker, Wissembourg, France). The chemical shifts were calculated with respect to (CH_3_)_3_Si(CH_2_)_3_SO_3_Na dissolved in D_2_O for NMR shift control. The spectra were taken at 62.90 MHz. The spectra (10,000 transients) were attained on a Bruker MSL300 FT-NMR spectrometer, at a frequency of 62.9 MHz. Chemical shifts were calculated relative to (CH_3_)_3_Si (CH_3_)_3_SO_3_Na in D_2_O, with a precision of 1 ppm. The relaxation delay was 5 s. 

#### 2.2.8. Matrix-Assisted Laser Desorption Ionization Time-of-Flight (MALDI-TOF) Analysis

The samples were treated with a NaCl solution (1.5 µL of 0.1 M) in a methanol/water mixture (1:1) to increase ion formation, and a drop was placed on the MALDI target (3 mm diameter) steel plate and dried. The samples and the matrix were then mixed in equal amounts, and 1.5 µL of the resulting slurry was placed on the MALDI target and dried at 40 °C for 2 h before being analyzed. A matrix of 2,5-dihydroxy benzoic acid was used. Red phosphorous (500–3000 Da) was used as a reference for spectrum calibration. Finally, after evaporation of the solvent, the MALDI target was introduced into the spectrometer. 

The spectra were recorded on a KRATOS AXIMA Performance mass spectrometer from Shimadzu Biotech (Kratos Analytical Shimadzu Europe Ltd., Manchester, UK). The irradiation source was a pulsed nitrogen laser with a wavelength of 337 nm. The length of one laser pulse was 3 ns. Measurements were carried out using the following conditions: positive polarity, a linear flight path, 20 kV acceleration voltages, and 100–150 pulses per spectrum. The delayed extraction technique was used applying delay times of 200–800 ns. The software MALDI-MS (Kratos Analytical Shimadzu Europe Ltd., Manchester, UK). as used for the data treatment. The oligomers can appear in the spectra either corresponding to their molecular weight or to their molecular weight +23 Da of the Na+ ion derived from the NaCl used as an enhancer. The spectra precision was ±1 Da.

## 3. Results and Discussion

### 3.1. Small-Scale Experiments on Tannin Extraction 

Table 1, Table 2 and Table 3 show the amounts of tannins extracted, non-tannins, catechin number or Stiasny number, which reflects the percentage of total polyphenolic material, the part rich in condensed tannins [27], tannin purity and extraction rate for the three fractions, C, M and F of ground pods of A. nilotica subsp. tomentosa. The results achieved by fraction C are summarized in Table 1. The tannin percentage and catechin number (6.42% and 4.7%, respectively) were achieved by 70GPC6. This means that a temperature of 70 °C and water-to-pod ratio of 6:1 were optimal parameters for the extraction process. Dentinho et al. [28] used a high ratio (10:1); however, there was no explanation of the effect of the ratio used on the amount of tannin extraction. They stated that the solvent should completely cover the bark and continuous mixing helps the process of extraction [28,29]. It is worth noting that when the surface area of both the solvent and extracting materials increased, it increased the amount extracted material. This could further be aided by continuous mixing of the solvents with the particles, which is possible only if a high solvent-to-solid ratio is used. This may also depend on the duration of the extraction; a longer duration like the one used for this study, which was overnight, may cause the solvent to destroy the cell structure, leading to an increase in the tannin yield [30].

The extraction rate was also higher while the tannin purity remained the same under all conditions used. Nevertheless, the results achieved by this fraction under the three conditions used was higher than the tannin percentage achieved by Petchidurai et al. and Shi et al., when they extracted tannins with 70% acetone containing 0.01% ascorbic acid using a Soxhlet method and kept their samples in a water bath at 50 °C for 10 h [31,32]. The water extraction was found to be more efficient than when 90% ethanol and 200 mL of glacial acetic acid were used as solvents at an extraction temperature of 70 °C for 2 h, which gave a high percentage of tannins [33]. It is worth noting that the high solubility of the tannins in water, which resulted in a high yield, compared to alcohols and other solvents could be interpreted by the high polarity of the water, which was apparently higher than when ethanol and acetone were used [34,35].

Table 2 shows the results achieved by fraction M. Again, the highest tannin percentage (17.3%) and catechin number (13.5) were obtained at a temperature of 70 °C and pod/water ratio of 1:6. It was noticed that the tannin percentage, catechin number and extraction rate were higher in fraction C. This observation is significant as it shows the effect of the fraction size on the extraction process.

Fraction F, as shown in Table 3, gave the highest percentage of tannin extract (43.7%) with a Stiasny number of 27.7% when the extraction temperature was 70 °C and water-to-pod ratio was 6:1 (70GGrF6; Table 3). The extraction rate was extraordinary (11.12), and the tannin purity (0.9) was significantly high as well. These results were also in line with what Antwi-Boasiako [36] achieved when he ground the bark of three hardwoods to a particle size of 0.5 µm and used hot water (70–90 °C) to extract the tannins; the catechin number was the highest.

The effect of using a fine particle size coupled with a high temperature and water-to-pod ratio was clearly manifested by this fraction. It has been observed that tannin extraction was found to be quicker when using smaller particle sizes as solvent could penetrate easily and shorten the extraction time [7]. From Table 1, Table 2 and Table 3, it can be deduced that the fraction size has a tremendous effect on the extraction process; the higher the tannin percentage, the higher the catechin number and tannin purity. Tannin analysis of the extracts for total solids, total solubles (sugars, gum and tannins) [37], non-tannins and catechin number (condensed tannins) (Table 1, Table 2 and Table 3) confirmed the quantitative data and revealed that all extracts obtained from oven-dried fractions C, M and F at different extraction conditions contained more than 10% tannins; this quantity is of commercial interest. It is worth noting that water tannin extraction was found to yield a high percentage of tannins compared to water/acetone and other solvents [38]. In addition, the optimum conditions for tannin extraction were identified as follows: the tannins containing materials should be ground to a fine particle size of 1 mµ, with a water-to-material ratio of 1:6 and temperature of 70 °C. Another important observation is that the tannin extraction rate ranged from 1.6 to 2.9 for both F and M extracts; it is higher than 1.5–2.0%, the rate of commercial interest. These conditions were used to scale up the extraction process in order to again confirm their feasibility and to have insight for commercial-scale production using water without any added chemicals. The extraction rate ranged from 1.6 to 2.9 for both F and M extracts, while it was lower for all C fraction extracts (small scale).

### 3.2. Scaled-Up Tannin Extraction Experiments

The results in Table 4 again confirm that high percentages of tannins and catechin number (47.60% and 57.50%, respectively) were obtained at 70 °C with a 6:1 water-to-pod ratio for the three fractions in the liquid and solid (spray-dried powder) phases. The obtained results were higher than what was reported in the literature when ethanol was used as a solvent [39,40]. It is interesting to note that tannin contents vary with the variation in species, age, and type of tissue [41]; however, the method of extraction and solvent used also have a great influence on the amount on tannins extracted [42]. The amounts of tannins, catechin number, non-tannins, total solids and solubles were increased in accordance with the increase in the amounts of extraction solvent and materials. The results confirmed the efficiency of 70GPF6, which produced the best results. They also confirmed that spray drying increased the percentage of tannins and catechin number [5,43], enhancing the concentration of tannins for all the fractions used and under all temperatures and water-to-materials ratios. This suggests a preference for work with the solid phase [3]. The tannin purity, i.e., the ratio of tannin to soluble solids, another quality criterion, was 0.6 or higher in all liquid extracts, while it decreased in the solid phase. Thus, most of the extracts were found to be attractive for commercial extraction of tannins.

### 3.3. Chemical Analysis of ANT and ASS Tannins

Table 5 summarizes the results of chemical analysis of the tannins extracted from the bark. It is important to note than the percentages of tannins for ANT (45.70%) and ASS (57.60%) were higher than the values achieved by the spray-dried powder of 70GPF6. The bark is considered the richest part of the plant in terms of tannin content [5]. However, the values reported for both ANT and ASS were lower than the values achieved in previous research [18,44] when the temperature was raised to 90 °C while other parameters were kept the same. Of the total polyphenolic materials, parts rich in condensed tannins were also high for both ANT and ASS tannins (65.01 and 69.20%, respectively). It has been observed that at 70–75 °C, the total extract percentage yield is lower. However, the proportion of polyphenols is much higher; thus, the tannin extract could be of much better quality for application in wood adhesives [3], which is the aim of this study. Solubility of materials also represents an important criterion for the tannin chemical analysis as it indirectly indicates the amount of tannins and their purity. It is higher for both of the acacia species. Thus, it is expected that both species could have a very good tannin quality; however, this is still to be confirmed by further analysis such as ^13^C NMR and MALDI-TOF.

### 3.4. Tensile Strength (TS) of Resin Preparations

Table 6 shows the results of the tensile strength (TS) achieved by the adhesives prepared from both phases of the three ground-pod fractions and urea formaldehyde resin (UF), which serves as a control. It has been observed that the TS values obtained from the three fractions in the liquid and solid phases exceeded the minimum requirement set by EN314-2 for interior use, i.e., 1.0 MPa [45]. The values achieved by the liquid phase for 70GPC6 and 70GPM6 were comparable to the value achieved by the UF resin. It is interesting to note that the value achieved by 70GPF6 in the liquid phase exceeded the TS of the UF. In the solid phase, the TS values for the three fractions exceeded TS of UF with 70GPF6 achieving the highest value. This again confirms the influence of the fine particle size on the extraction process. It is very important to note that the resin formulations prepared in this study were able to produce excellent bonding properties that exceeded the standard and UF resin. It also confirmed that the tannin–phenolic resin was able to penetrate the wood and cure within the wood cells after heat was applied to the adhesive system [46].

### 3.5. ^13^C NMR Analyses

**Figure 1:**
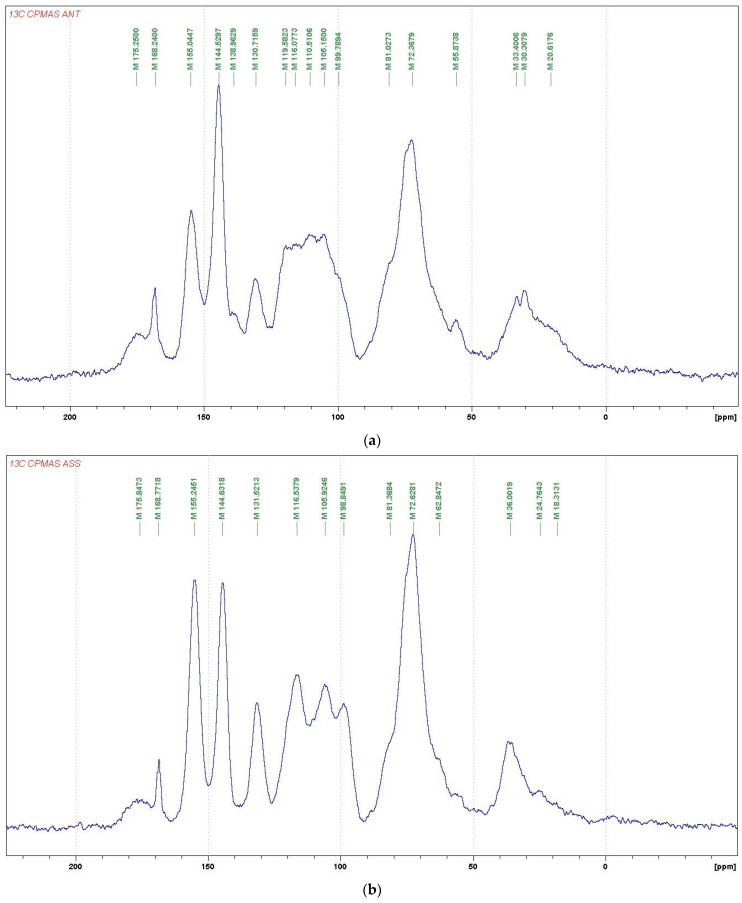
Comparison of ^13^C NMR spectra of ANT (**a**) and ASS (**b**) tannin extracts.

For further investigations on the structure of the condensed tannins, ^13^C NMR analysis was employed. The ^13^C NMR spectra obtained (Figure 1) for *Acacia nilotica tomentosa* (ANT) and *Acacia seyal* var. *seyal* present information on the nature of their tannin extracts. The two spectra show typical signals due to the presence of condensed tannins, containing a majority of procyanidins and prodelphinidins. The signals at 155 ppm are assigned to the C5, C7 and C9 of flavonoid units to which an oxygen atom is linked, namely –OH groups for C5 and C7 and the C-O-C group of the heterocycle for C9, indicating a great majority of flavonoid unit A-rings with two –OH groups. The signal at 131 ppm is assigned to the C1’ of the flavonoid units. The signals at 116 ppm (C2’, C5’), 120 ppm (C6’), and 145 ppm (C3’, C4’) show the presence of catechin/epicatechin units with the 116 ppm having been more marked for ASS than for ANT in relation to the 120 ppm shift, showing the lower importance of the free C6’ on the unit B-rings. The sharp and high signal at 145–146 ppm is typical of the presence of prodelphinidins (gallocatechin/epigallocatechin). The procyanidin/prodelphinidin ratio of condensed tannins is usually determined from the relative ratio of these two peaks. According to the intensity of the peaks presented in Table 7, the procyanidin/prodelphinidin ratios were approximately 70%/30% for ANT and 60%/40% for ASS. The signals at 110 and 105 ppm are characteristics of the C4–C8 and C4–C6 interflavonoid bonds. For ANT, it appears that the proportions of the two types of interflavonoid bonds are approximately equal, while for ASS, the C4–C6 bond appears to predominate.

The region between 30 and 90 ppm is due to the signals of C2, C3, and C4 in flavan- 3-ol units. The two signals at 72 ppm indicated the presence of 2,3-cis and at 81 ppm to 2,3-trans isomers. The spectrum demonstrated that both of the stereoisomers co-exist in the two tannins. However, in ANT, there appears to be a predominance of the transform stereoisomers based on intensity.

### 3.6. MALDI-TOF Analyses

The MALDI-TOF analysis assignments in Table 8 show that the two Acacia species, ANT (Figure S1 Supplementary Material) and ASS (Figure S2 Supplementary Material), present similar oligomer types and distributions. They are mainly procyanidin–prodelphinidin tannins, as also shown in the past by gel time studies [18]. However, there are two interesting considerations on both tannins that have not been noted before. First, there are glucose (and or mannose)-based short carbohydrate chains present that are sometimes covalently linked to flavonoid units. This was already observed for the first time in other tropical species [47] with the occasional presence of glucuronic acid either due to the extraction procedure or MALDI analysis. There are also loose short carbohydrate chains that might derive just from fragments of hemicelluloses generated during the tannin extraction. Second, and more unusually, there are some rare traces of hydrolysable tannins that have been extracted with the bulk of condensed ones. These are small in proportion if one has to judge from the peak heights and include the oligomers at 436 Da, 570 Da and 657 Da, the first two in ANT and the last one in ASS, the latter one even more clearly derived from hydrolysable tannin [47]. These species are obtained by stripping galloyl residues from pentagalloylglucose and its oligomers [48]. From Table 9, the intensities of the peak of procyanidin/prodelphinidins were found to be 81/19% for ANT and 71/29% for ASS. Thus, both tannin compositions are mainly composed of procyanidins, confirming the results obtained by the ^13^C NMR. To conclude, the results obtained from the analysis of both techniques refuted the information provided by some researchers that tannin hot water extraction generated only monomers; therefore, it is impossible to elucidate the tannin structure from hot-water-extracted solutions [15].

## 4. Conclusions

The current research work investigated the effect of parameters such as the particle size, solid/solvent ratio, and temperature on the tannin extraction process in order to develop cost-effective methods that offer better environmental sustainability and structural performance for Acacia species. Among the three studied fractions (C, M and F), the fine fraction gave the best results with regard to the tannin and condensed tannin amount. The effective water-to-pod ratio was 1:6 with all fractions used; however, the best results were achieved with the fine fraction at 70 °C, which appeared to be the most efficient extraction temperature compared to 30 and 50 °C. Adhesives produced by these tannins showed high tensile strength that conformed with the EN for veneers and plywood applications. The results of ^13^C NMR and MALDI-TOF of tannins extracted from the bark of ANT and ASS confirmed the efficiency of water as the optimum solvent for tannin extraction; the spectra showed the presence of monomers as well as oligomers of the condensed tannins.

The results obtain using ^13^C NMR and MALDI-TOF showed that the bark of ANT and ASS contains procyanidin, a reactive type of tannin suitable for use as an adhesive in the wood industry. Hot water for tannin extraction is an efficient solvent; however, parameters such as the fraction size, temperature, and solvent/solid ratio should be optimized and adopted. Future work targeting the optimization of these parameters will be planned for further investigation. More investigations into the fine fraction and spray drying parameters will also be carried out in the future.

## Figures and Tables

**Table 1:** Results of tannin chemical analysis and extraction conditions of the course fraction (C) of the ANT pods with water.

Extraction Temperature (°C)	30			50			70		
Water-to-pod ratio	2:1	4:1	6:1	2:1	4:1	6:1	2:1	4:1	6:1
Moisture content (%)	7.20			6.50			6.48		
Tannins (T) %	2.83	4.39	5.03	2.17	5.49	5.64	1.44	4.41	6.42
Catechin number	1.97	2.61	3.27	1.63	2.82	3.73	1.78	3.49	4.70
Non-tannins (NT)	2.99	5.15	5.76	2.34	3.96	5.49	2.38	4.22	5.70
Total solids %	6.43	10.63	12.32	5.59	9.85	11.61	4.56	9.8	11.95
Total solubles (Tso) %	5.83	9.55	11.78	4.51	9.42	11.34	3.82	8.63	10.92
Purity (T/Tso)	0.5	0.5	0.5	0.5	0.51	0.5	0.4	0.5	0.5
Extraction rate (T/NT)	0.95	0.85	0.87	0.93	0.22	0.98	0.60	1.04	1.1
pH	4.26	4.30	3.81	3.81	4.09	4.20	4.28	3.78	4.08

C ≥ 2 mm.

**Table 2:** Results of tannin chemical analysis and extraction condition of the medium fraction (M) of the ANT pods with water.

Extraction Temperature (°C)	30			50			70		
Water-to-pod ratio	2:1	4:1	6:1	2:1	4:1	6:1	2:1	4:1	6:1
Moisture content (%)	6.2			6.19			6.29		
Tannins (T) %	9.59	12.34	15.08	8.66	14.82	13.76	9.59	12.51	17.29
Catechin number	8.79	12.26	13.08	6.79	12.19	13.33	8.08	10.79	13.46
Non-tannins (NT)	8.79	10.74	11.03	8.03	4.06	13.39	6.44	7.03	6.92
Total solids %	24.83	31.48	34.52	17.85	29.14	29.8	21.3	27.4	32.31
Total solubles (Tso) %	11.39	23.08	26.11	16.69	25.88	27.16	16.54	19.53	24.26
Purity (T/Tso)	0.5	0.5	0.6	0.5	0.6	0.5	0.6	0.6	0.7
Extraction rate (T/NT)	1.14	1.15	1.37	1.08	1.30	1.03	1.38	1.78	2.5
pH	3.57	3.88	4.10	3.44	3.59	3.45	4.30	4.34	3.37

M = 1 µm.

**Table 3:** Results of tannin chemical analysis and extraction conditions of the fine fraction (F) of the *A. nilotica* ssp. *tomentosa* pods with water.

Extraction Temperature (°C)	30			50			70		
Water-to-pod ratio	2:1	4:1	6:1	2:1	4:1	6:1	2:1	4:1	6:1
Moisture content (%)	6.47			7.44			7.45		
Tannins (T) %	24.49	24.61	28.52	17.04	24.59	24.49	41.92	34.77	43.69
Catechin number	17.69	22.75	24.62	18.36	23.19	21.86	26.42	24.78	27.67
Non-tannins (NT)	13.74	21.13	11.34	16.39	14.08	21.79	8.54	8.45	3.93
Total solids %	39.09	52.09	51.82	35.28	44.58	49.57	50.88	53.16	53.94
Total solubles (Tso) %	40.81	45.74	39.86	33.43	40.11	46.39	50.46	42.83	47.62
Purity (T/Tso)	0.6	0.5	0.7	0.5	0.6	0.6	0.8	0.8	0.9
Extraction rate (T/NT)	1.78	1.65	2.61	2.51	1.09	1.85	4.9	4.33	11.12
pH	3.80	3.93	3.93	3.52	3.52	3.56	4.32	3.71	4.01

F ˂ 1 µm.

**Table 4:** Results of chemical analysis of the tannins extracted from the three fractions of ANT pods (liquid) and spray drying (solid).

Tannins Extract Code	70GPC6		70GPM6		70GPF6	
Phase	Liquid	Solid	Liquid	Solid	Liquid	Solid
MC (%)	6.23	5.09	6.35	4.36	5.21	5.15
Tannins (T) %	10.2	38.9	16.4	46.8	32.5	47.6
Catechin number	9.0	45.7	16.5	52.8	30.7	57.2
Non-tannins (NT) %	6.30	46.19	16.40	34.40	10.90	41.50
Total solids %	16.8	85.9	31.4	89.8	46.6	92.3
Total solubles (Tso) %	16.5	85.9	26.3	81.2	43.4	81.4
Purity (T/Tso)	0.70	0.50	0.60	0.50	0.75	0.60
Extraction rate (T/NT)	1.60	0.80	1.70	1.00	2.98	1.15

**Table 5:** Chemical composition of the bark extract of ANT and ASS.

Subspecies	Tannins (T%)	Total Polyphenolic Materials (%)	Soluble Materials (SM%)	Tannin Purity (T/SM)
ANT	50.7	65.01	81.3	0.6
ASS	57.6	69.20	92.6	0.6

**Table 6:** Veneers with different resins prepared from the three fractions and Mimosa tannins.

Sample#	Tannin Extract Fraction	Tensile Strength Liquid Phase (MPA)	Tensile Strength Solid Phase (MPA)
1	70GPC6	1.33	1.65
2	70GPM6	1.55	1.88
3	70GPF6	1.70	2.48
4	UF	1.60	

**Table 7:** Relative shift peak intensities for the three polyflavonoid-type tannins derived from the ^13^C NMR analysis (%).

			B-Ring				Interflavonoid Bond			Free	C Trans		Free
			Catechol	Pyrogall									
	C5,C7 C9	C3’ C4’ PD	C′1	C1’	C′6	C′5, C′2 PC	C4–C8	C4–C6	C10	C6,C8		C3 cis	C4
NMR shifts ppm	160–155	145–148 PD	131–129	132–135	123–121	120–116	115–110	105	103	97–98	83–86	71–68	28
Ant	60	100	40	50	33	52	50	25	25	33	25	82	35
ASS	86	85	48	-	-	56	43	53	-	48	15	100	32

**Table 8:** Assignment of oligomer constituents for the extraction of ANT and ASS.

272 Da = fisenidin, no Na+ (ASS and ANT)
290 Da = catechin or robinetinidin, no Na+ (ASS and ANT)
294 Da = fisetinidin, with Na+ (ASS and ANT)
304 Da = gallocatechin (delphinidin), no Na+, deprotonated (ASS and ANT)
316 Da = catechin or robinetinidin, with Na+, multiprotonated (ASS and ANT)
326–328 Da = gallocatechin (delphinidin), with Na+, protonated (328 Da) (ASS and ANT)
346 Da = fragment, no Na+ (346 Da) and with Na+, and with Na+ (367.9 Da) (ASS and ANT)
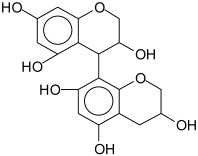
364 Da = glucose dimer with Na+ (ASS and ANT)
436 Da = 1 Galloyl residue on 2 stripped glucose residues, dimer (ANT)
459 Da = fisetinidin-glucose, with Na+, protonated (ASS)
503.8 Da = glucose trimer, no Na+, and 525 Da with Na+ (ASS)
519–521 Da = glucose-glucose-glucuronic acid, no Na+, and 541.7 Da with Na+ (ASS and ANT)
543.7 Da = fisetinindin dimer, no Na+, and 567 Da with Na+ (ASS)
561–562 Da = catechin-fisetinidin dimer, no Na+, or robinetinidin-fisetinidin dimer, no Na+, and 583.6 Da with Na+ (ASS and ANT)
570 Da = 1 Galloyl residue on 3 glucoses chain, trimer (ANT)
601 and 604 Da = catechin dimer, with Na+ (ASS and ANT)
613 Da = catechin-glucose-glucose, no Na+ (ASS)
617–619 Da = gallocatechin-catechin dimer, with Na+ (ASS and ANT)
657 Da = Trigalloyl glucose, monomer (ASS)
721 Da = catechin-fisetinidin-glucose, no Na+, deprotonated (ASS and ANT)
727 Da = fisetinidin-fisetinidin-glucose, with Na+, deprotonated (ASS and ANT)
742–743 Da = Catechin fisetinidin-glucose, with Na+ (ASS and ANT)
756 Da = Catechin-fisetinidin-glucuronic acid, with Na+ (ASS)
759 Da = gallocatechin-fisetinidin-glucose, no Na+ OR/AND catechin-catechin-glucose, no Na+ (ASS)
885–887 Da = catechin trimer with Na+, deprotonated (ASS and ANT)
903 Da = gallocatechin-catechin-catechin trimer with Na+, deprotonated (ASS and ANT)
919 Da = gallocatechin-gallocatechin-catechin trimer, with Na+ (ASS and ANT)
935 Da = gallocatechi-gallocatechin-gallocatechin trimer with Na+ (mainly ASS; and ANT)
1055 Da = catechin trimer-glucose, with Na+ (ASS)
1175–1176 Da = Catechin tetramer, with Na+, deprotonated (ASS and ANT)
1191–1192 Da = gallocatechin-(catechin)_3_ tetramer with Na+, deprotonated (ASS and ANT)
1479–1480 Da = gallocatechin-(catechin)_4_ pentamer with Na+, deprotonated (ASS and ANT)
1496 Da = (gallocatechin)_2_-(catechin)_3_ pentamer, with Na+, deprotonated (ANT)

**Table 9:** Catechin and gallocatechin dimer mass range and peak intensity for ANT and ASS.

Dimer Types	Experimental Intensities (%)	
Tannins	Catechin dimers (601–603 Da)	Gallocatechin dimers (628–667 Da)
ANT	81	19
ASS	71	29

## Data Availability

Data are contained in this article and its Supplementary Material.

## Supplementary Materials

The following supporting information can be downloaded at https://www.mdpi.com/article/10.3390/polym16111550/s1: Figure S1. MALDI ToF spectra of ANT: (a) 250–2000 Da range; (b) 250–300 Da range; (c) 300–400 Da range; (d) 400–600 Da range; (e) 600–800 Da range; (f) 800–1000 Da range; (g) 1000–2000 Da range. Figure S2. MALDI ToF spectra of ANT: (a) 250–1000 Da range; (b) 250–300 Da range; (c) 300–400 Da range; (d) 400–600 Da range; (e) 600–800 Da range; (f) 800–1000 Da range; (g) 1000–2000 Da range.
