# Supplementary material for: Effect of Technological Factors on the Extraction of Polymeric Condensed Tannins from Acacia Species
Source: Polymers (Basel). 2024 May 30;16(11):1550. doi: 10.3390/polym16111550 (PMC11174908; doi:10.3390/polym16111550)
Supplement: Supplementary file 1 [file polymers-16-01550-s001.zip › polymers-3020175-supplementary.pdf]

SUPPLEMENTARY MATERIALS

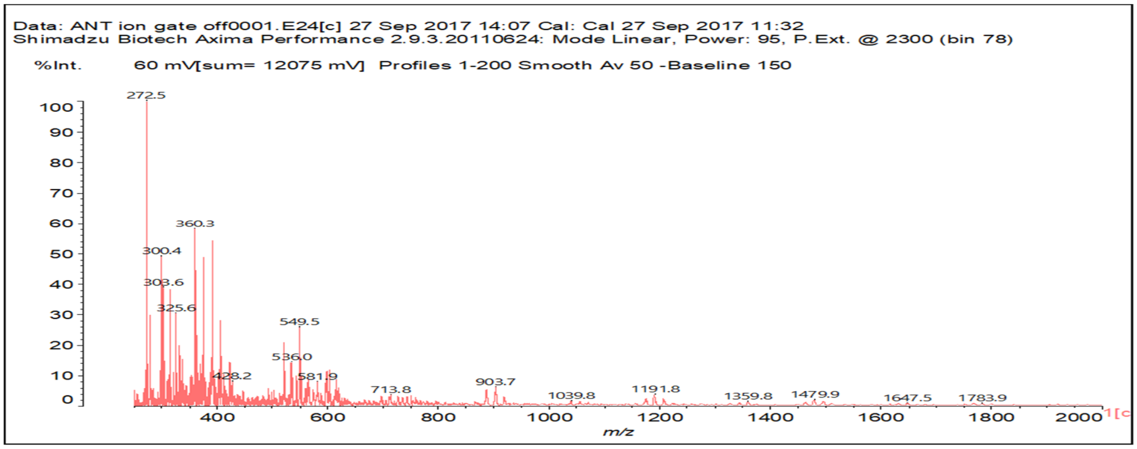

(a)

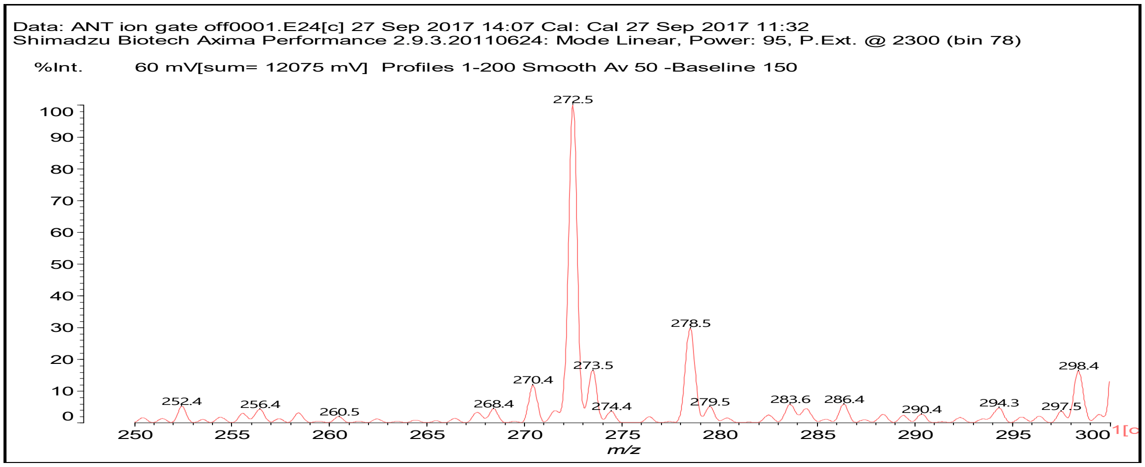

(b)

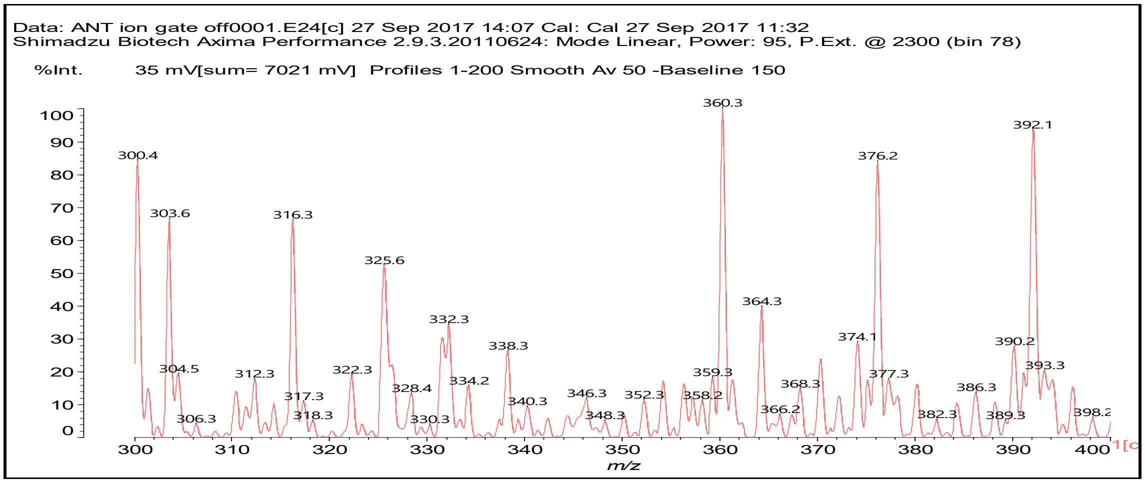

(c)

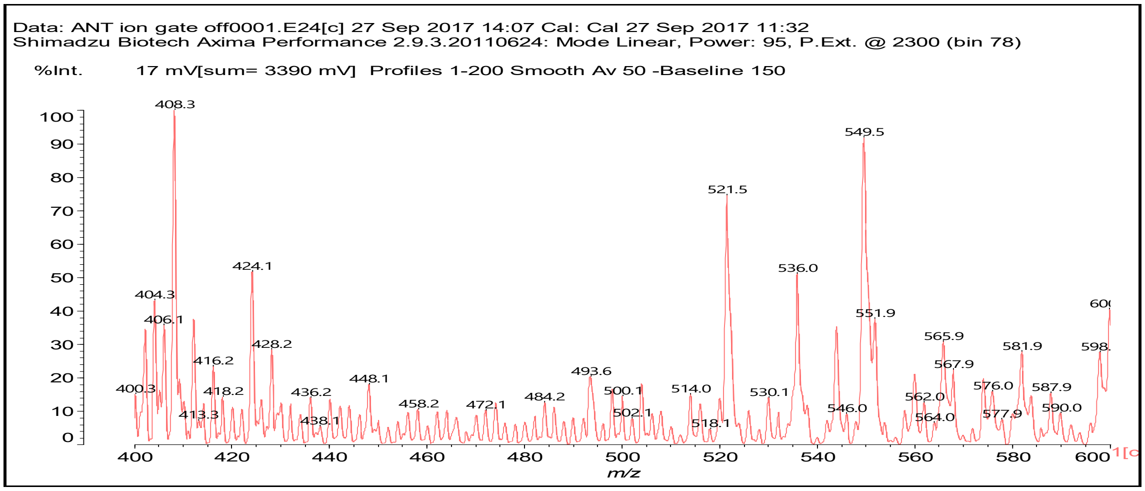
Data: ANT ion gate off0001.E24[c] 27 Sep 2017 14:07 Cal: Cal 27 Sep 2017 11:32
Shimadzu Biotech Axima Performance 2.9.3.20110624: Mode Linear, Power: 95, P.Ext. @ 2300 (bin 78)
%Int.
17 mV[sum= 3390 mV] Profiles 1-200 Smooth Av 50 -Baseline 150
408.3
549.5
521.5
536.0
424.1
404.3
406.1
551.9
428.2
565.9
581.9
416.2
567.9
493.6
448.1
400.3
418.2
500.1
514.0
530.1
576.0
587.9
562.0
458.2
472.1
484.2
413.3
436.2
438.1
502.1
518.1
546.0
564.0
577.9
590.0
m/z

(d)

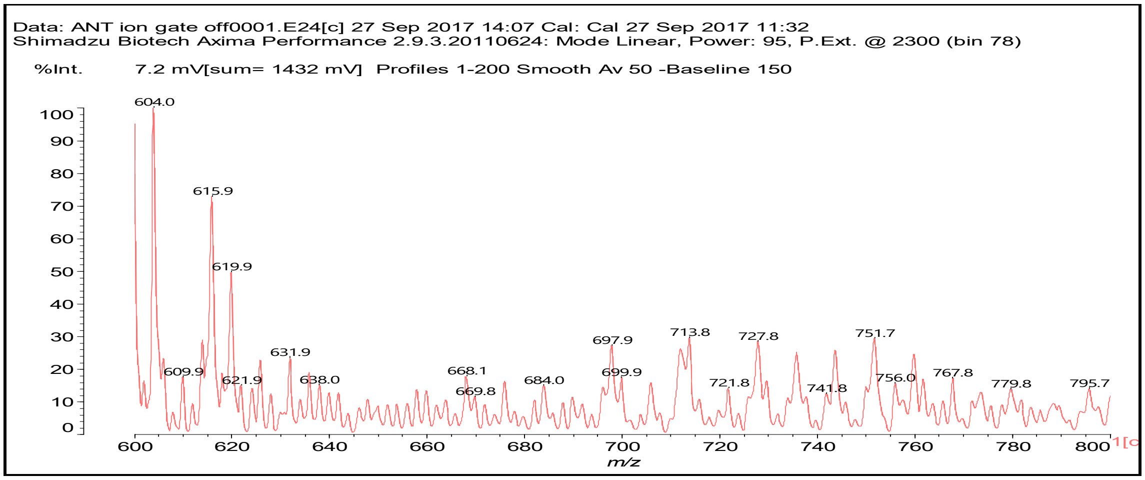
Data: ANT ion gate off0001.E24[c] 27 Sep 2017 14:07 Cal: Cal 27 Sep 2017 11:32
Shimadzu Biotech Axima Performance 2.9.3.20110624: Mode Linear, Power: 95, P.Ext. @ 2300 (bin 78)
%Int.
7.2 mV[sum= 1432 mV] Profiles 1-200 Smooth Av 50 -Baseline 150
604.0
615.9
619.9
631.9
697.9
713.8
727.8
751.7
609.9
621.9
638.0
668.1
669.8
684.0
699.9
721.8
741.8
756.0
767.8
779.8
795.7
m/z

(e)

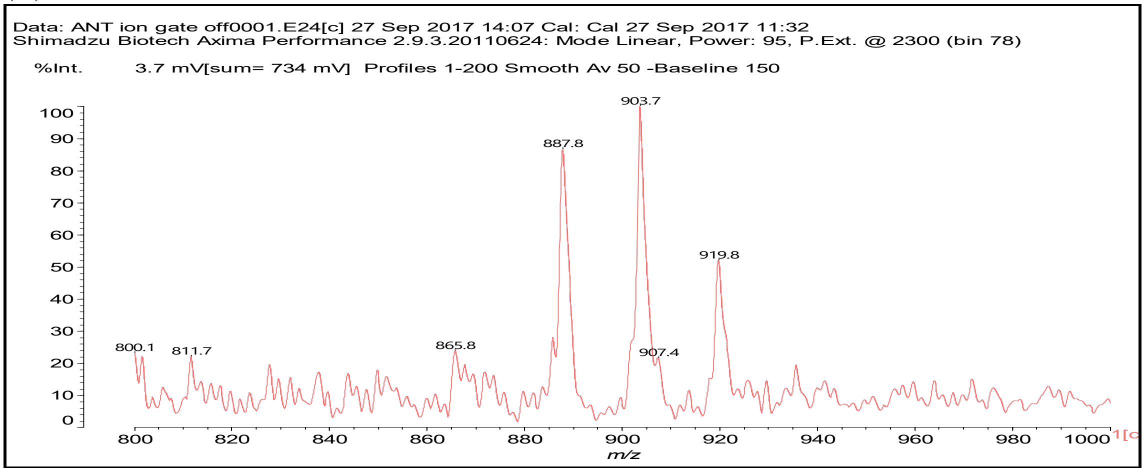
Data: ANT ion gate off0001.E24[c] 27 Sep 2017 14:07 Cal: Cal 27 Sep 2017 11:32
Shimadzu Biotech Axima Performance 2.9.3.20110624: Mode Linear, Power: 95, P.Ext. @ 2300 (bin 78)
%Int.
3.7 mV[sum= 734 mV] Profiles 1-200 Smooth Av 50 -Baseline 150
903.7
887.8
919.8
800.1
811.7
865.8
907.4
m/z

(f)

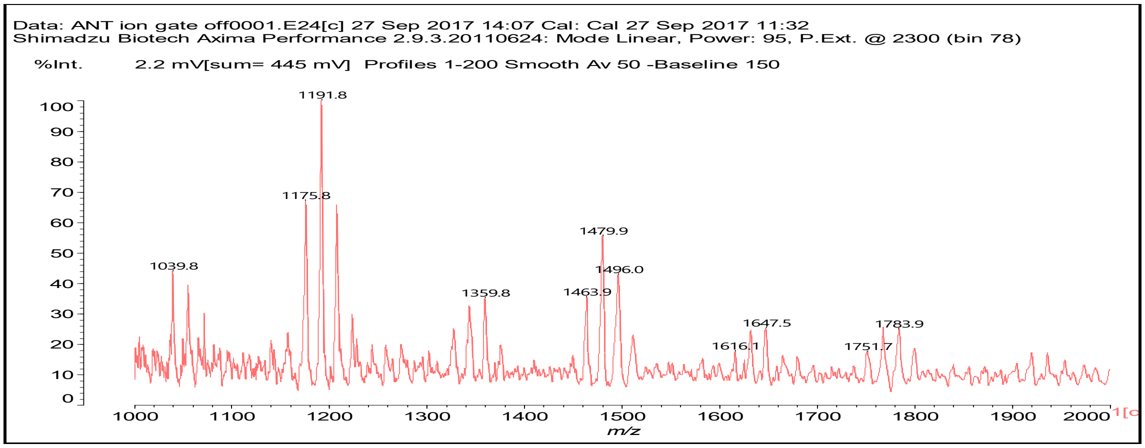

(g)

Figure S1. MALDI ToF Spectra of ANT (a) 250-2000 Da range ; (b) 250-300 Da range ; (c) 300-400 Da range ; (d) 400-600 Da range; (e)600-800 Da range; (f) 800-1000 Da range; (g) 1000-2000 Da range.

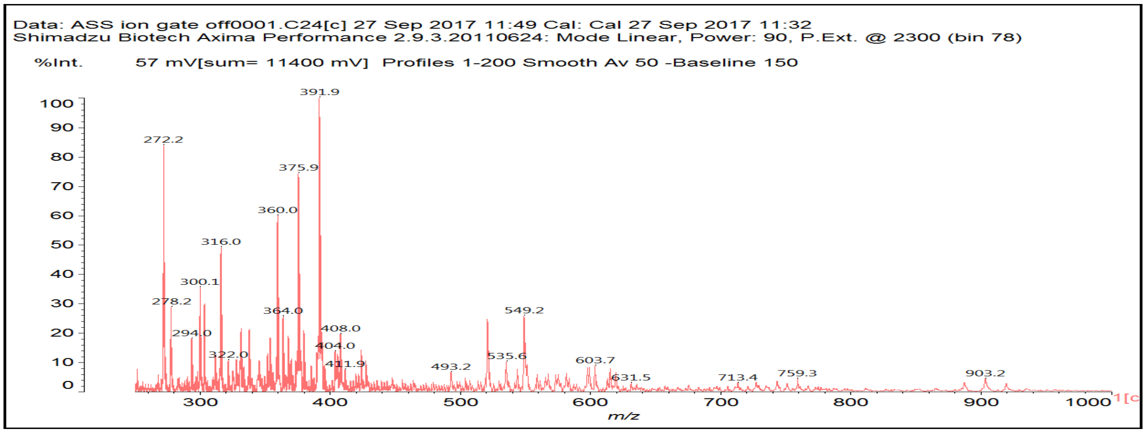

(a)

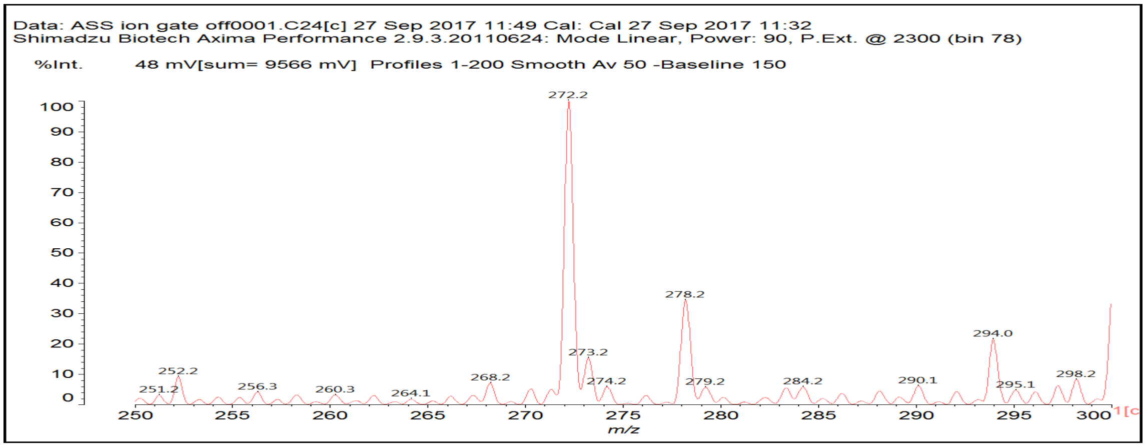

(b)

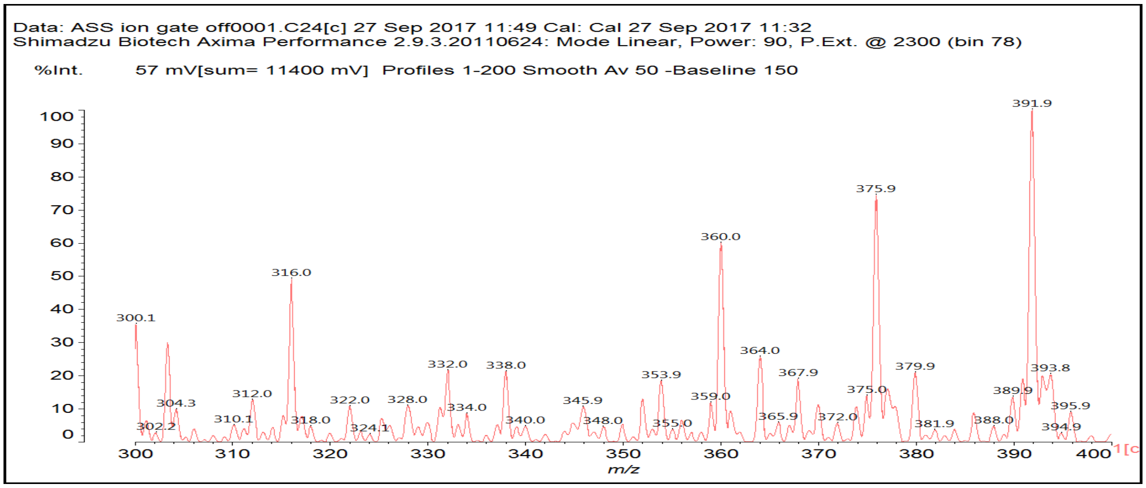
Data: ASS ion gate off0001.C24[c] 27 Sep 2017 11:49 Cal: Cal 27 Sep 2017 11:32
Shimadzu Biotech Axima Performance 2.9.3.20110624: Mode Linear, Power: 90, P.Ext. @ 2300 (bin 78)
%Int. 57 mV[sum= 11400 mV] Profiles 1-200 Smooth Av 50 -Baseline 150
m/z

(c)

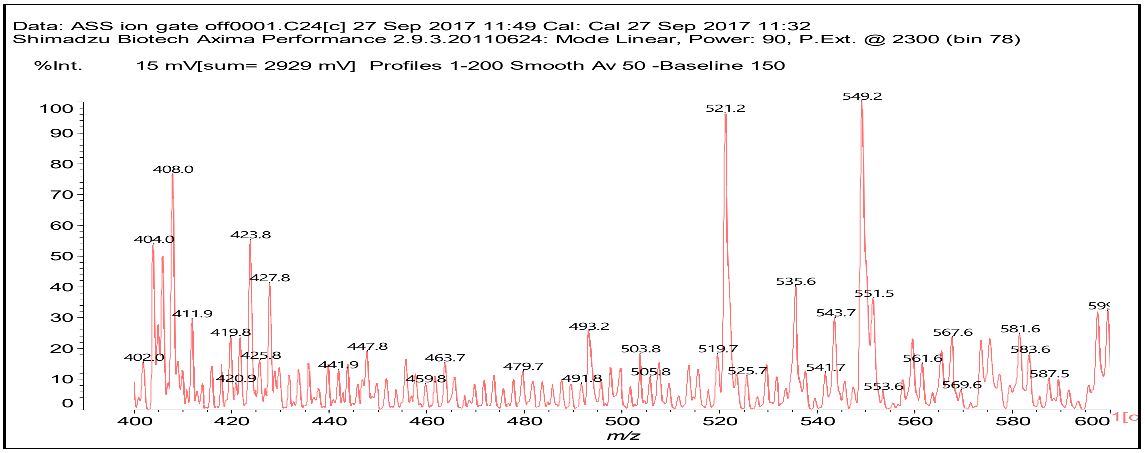
Data: ASS ion gate off0001.C24[c] 27 Sep 2017 11:49 Cal: Cal 27 Sep 2017 11:32
Shimadzu Biotech Axima Performance 2.9.3.20110624: Mode Linear, Power: 90, P.Ext. @ 2300 (bin 78)
%Int. 15 mV[sum= 2929 mV] Profiles 1-200 Smooth Av 50 -Baseline 150
m/z

(d)

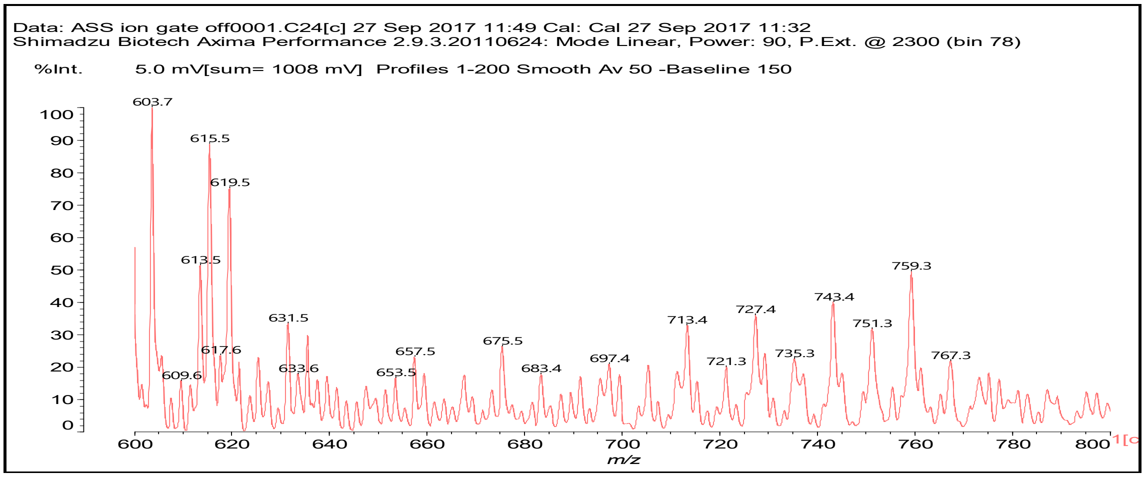
Data: ASS ion gate off0001.C24[c] 27 Sep 2017 11:49 Cal: Cal 27 Sep 2017 11:32
Shimadzu Biotech Axima Performance 2.9.3.20110624: Mode Linear, Power: 90, P.Ext. @ 2300 (bin 78)
%Int. 5.0 mV[sum= 1008 mV] Profiles 1-200 Smooth Av 50 -Baseline 150
m/z

(e)

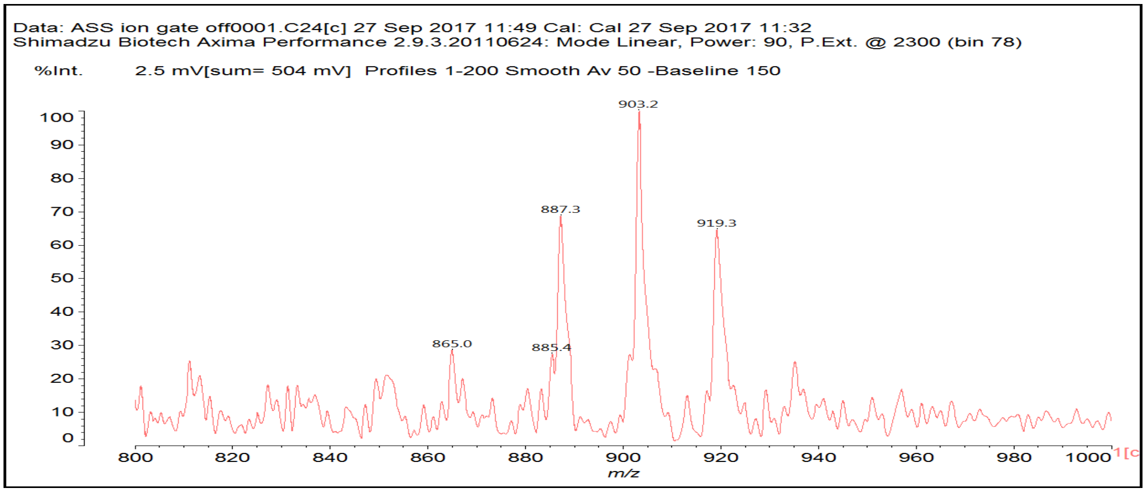

(f)

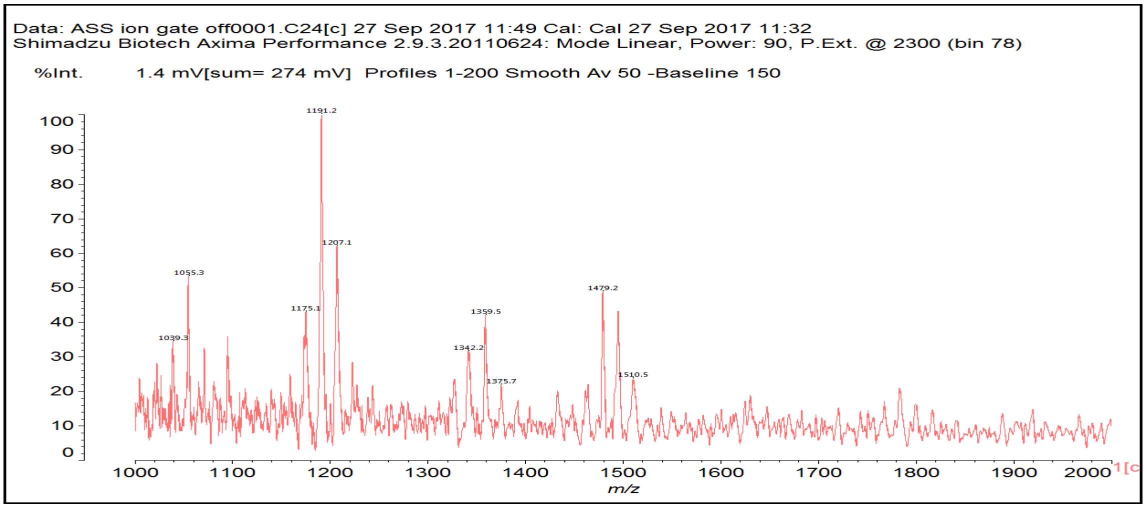

(g)

Figure S2. MALDI ToF Spectra of ANT (a) 250-1000 Da range ; (b) 250-300 Da range ; (c) 300-400 Da range ; (d) 400-600 Da range; (e)600-800 Da range; (f) 800-1000 Da range; (g) 1000-2000 Da range.
